# GFAP-Driven GFP Expression in Activated Mouse Müller Glial Cells Aligning Retinal Blood Vessels Following Intravitreal Injection of AAV2/6 Vectors

**DOI:** 10.1371/journal.pone.0012387

**Published:** 2010-08-24

**Authors:** Wendy M. Aartsen, Koen W. R. van Cleef, Lucie P. Pellissier, Robert M. Hoek, Rogier M. Vos, Bas Blits, Erich M. E. Ehlert, Kamaljit S. Balaggan, Robin R. Ali, Joost Verhaagen, Jan Wijnholds

**Affiliations:** 1 Department of Neuromedical Genetics, Netherlands Institute for Neuroscience, an Institute of the Royal Netherlands Academy of Arts and Sciences, Amsterdam, The Netherlands; 2 Department of Neuroregeneration, Netherlands Institute for Neuroscience, an Institute of the Royal Netherlands Academy of Arts and Sciences, Amsterdam, The Netherlands; 3 Amsterdam Molecular Therapeutics, Amsterdam, The Netherlands; 4 Division of Molecular Therapy, Institute of Ophthalmology, University College London, London, United Kingdom; Brigham and Women's Hospital, Harvard Medical School, United States of America

## Abstract

**Background:**

Müller cell gliosis occurs in various retinal pathologies regardless of the underlying cellular defect. Because activated Müller glial cells span the entire retina and align areas of injury, they are ideal targets for therapeutic strategies, including gene therapy.

**Methodology/Principal Findings:**

We used adeno-associated viral AAV2/6 vectors to transduce mouse retinas. The transduction pattern of AAV2/6 was investigated by studying expression of the green fluorescent protein (GFP) transgene using scanning-laser ophthalmoscopy and immuno-histochemistry. AAV2/6 vectors transduced mouse Müller glial cells aligning the retinal blood vessels. However, the transduction capacity was hindered by the inner limiting membrane (ILM) and besides Müller glial cells, several other inner retinal cell types were transduced. To obtain Müller glial cell-specific transgene expression, the cytomegalovirus (CMV) promoter was replaced by the glial fibrillary acidic protein (GFAP) promoter. Specificity and activation of the GFAP promoter was tested in a mouse model for retinal gliosis. Mice deficient for Crumbs homologue 1 (CRB1) develop gliosis after light exposure. Light exposure of *Crb1^−/−^* retinas transduced with AAV2/6-GFAP-GFP induced GFP expression restricted to activated Müller glial cells aligning retinal blood vessels.

**Conclusions/Significance:**

Our experiments indicate that AAV2 vectors carrying the GFAP promoter are a promising tool for specific expression of transgenes in activated glial cells.

## Introduction

Müller glial cells are the predominant glial cell type of the vertebrate retina. Müller glial cells span the entire thickness of the retina and are involved in a wide variety of physiological processes that are vital to proper functioning of the retinal neurons. They are important in retinal development, provide their neighboring neurons with metabolic support, maintain retinal ion and water homeostasis, contribute to the recycling of neurotransmitters as well as photopigments and protect the neurons against oxidative stress [1]. In addition to their functions in the healthy retina, Müller glial cells play crucial roles in almost all retinal pathologies, such as retinal detachment, diabetic retinopathy, inflammation and glaucoma [1], [2]. In response to retinal disease or injury, Müller glial cells are activated. This can eventually lead to gliosis. Activated Müller glial cells exert several neuroprotective activities that promote the survival of the retinal neurons during stress, such as the secretion of neurotrophic factors as well as antioxidants [3]. However, the dedifferentiated state of activated Müller glial cells also contributes to neuronal stress due to impairment of their neurosupportive activities [4]. Because of their crucial role in retinal disease, Müller glial cells are important targets for gene therapy [5].

Of the viral vectors that have been tested in retinal gene transfer, those derived from the adeno-associated virus (AAV) are regarded as most promising [6]. AAV vectors have been used extensively to transfer genes to the retina in many animal models for retinal diseases and shown to be useful for the treatment of genetic defects that affect either photoreceptor cells or retinal pigment epithelium (RPE) [7]. The efficacy and safety of AAV vectors has recently been demonstrated in Leber's congenital amaurosis (LCA) patients with *RPE65* mutations [8]–[11].

The goal of this study is to obtain a vector system that allows expression of transgenes only in activated Müller glial cells present at the site of retinal disease or injury. In rats, after intravitreal injection, AAV2/6 is able to transduce to equal extent retinal ganglion cells, amacrine, bipolar and Müller glial cells, whereas horizontal and photoreceptor cells were transduced to much lower extent [12]. When injected intravitreally, the inner limiting membrane (ILM) might act as a barrier to efficient transduction of the retina [13]. Depending on the time-point of administration, intravitreal delivery of AAV vectors results in transduction of a different set of retinal cell types. For example, more ganglion cells were transduced by AAV2/2 when delivered to the adult rat retina compared with delivery at P0 [14]. Moreover, the viral titer and the promoter used to drive the transgene have great influence on the level and pattern of expression [15]. All these aspects were considered in our study as we further optimized the transduction efficiency of AAV2/6 for mouse glial cells. Our results showed that AAV2/6 vectors were able to transduce mouse Müller glial cells aligning retinal blood vessels. Additionally, the ILM should be regarded as a barrier to intravitreally delivered AAV2/6, since collagenase treatment substantially increased the penetration of AAV2/6 into the entire retina. The consequence of better AAV2/6 penetration was the transduction of retinal ganglion cells, amacrine, horizontal, bipolar and Müller glial cells throughout the retina.

In reaction to retinal damage or cellular defects, retinal Müller glial cells become activated. This activation is characterized by the induction of glial fibrillary acidic protein (GFAP) and the downregulation of p27^kip1^ and cyclin D3 [2]. Previously, in transgenic mice, it has been shown that the GFAP promoter can drive green fluorescent protein (GFP) expression specifically in Müller glial cells and that this expression is induced once the cells are activated [16]. In this study, by incorporating the GFAP promoter instead of the commonly used cytomegalovirus (CMV) or CMV early enhancer/chicken β-actin (CAG) promoters into AAV2/6 vectors, we aimed to restrict transgene expression to activated Müller glial cells. The Müller glial cell-targeted gene transfer was tested in a mouse model deficient for Crumbs homologue 1 (*CRB1*) that exhibits retinal degeneration accompanied by Müller cell gliosis. *CRB1* encodes a transmembrane protein that is localized in Müller glial cells [17] at the subapical region (SAR) just above the adherens junctions (AJs) that form the outer limiting membrane (OLM). The AJs connect the Müller glial cells to the photoreceptor cells. CRB1 is a key component of a complex of scaffolding proteins involved in stabilization of these AJs [18]. It has been demonstrated in CRB1-deficient (*Crb1^−/−^*) mice that the absence of functional CRB1 leads to a loss of interaction between Müller glial cells and photoreceptor cells which causes retinal disorganization followed relatively late by retinal degeneration accompanied by Müller cell gliosis [19], [20]. The retinal degeneration observed in *Crb1^−/−^* mice corresponds to that seen in patients with *CRB1* mutations, although it is only observed in one quadrant of the retina [20]. In humans, mutations in *CRB1* are related to LCA and severe forms of retinitis pigmentosa (RP), which are characterized by progressive retinal degeneration causing blindness at birth or early adulthood, respectively [21], [22].

By transducing the *Crb1^−/−^* mouse retina using AAV2/6 vectors in combination with the GFAP promoter, we demonstrate that it is possible to specifically express transgenes in activated Müller glial cells, without expression in retinal ganglion cells, amacrine, bipolar, horizontal or photoreceptor cells. Transduced Müller glial cells started to express the GFP transgene when activated by ciliary neurotrophic factor (CNTF) or injury. These results suggest that AAV2/6 vectors carrying the GFAP promoter to drive the expression of neuroprotective or anti-angiogenic factors might be a promising tool to treat retinal disorders.

## Results

### Transduction efficiency of AAV2/6

**Figure 1:**
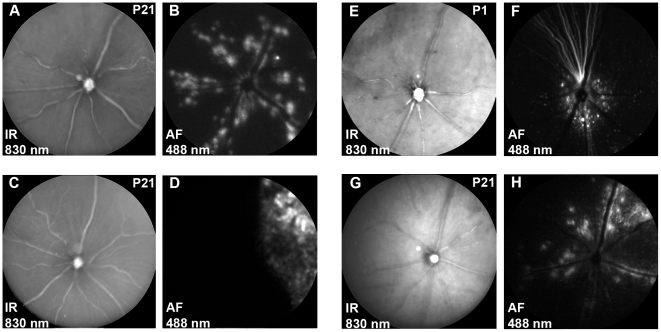
SLO of AAV2/6-CMV-GFP transduced wild-type mouse retinas 3 weeks post injection. SLO analysis was performed at 830 (IR) and 488 (AF) nm on all animals and representative examples are shown. After intravitreal injection of AAV2/6-CMV-GFP at postnatal day 21 (n = 6), the integrity of the retina appeared normal (**A**) and the fluorescent signal for GFP was detected along the major retinal blood vessels (n = 6) (**B**). After subretinal injection with AAV2/6-CMV-GFP at postnatal day 21 (n = 6), retinal integrity was not affected (**C**), while the GFP expression was detected at a restricted part of the retina (**D**). Intravitreal injection of AAV2/6-CMV-GFP at postnatal day 1 (n = 5) did no major damage to the retina (**E**) and GFP expression was concentrated at the center of the retina near the optic nerve (**F**). Intravitreal injection of AAV2/6-CAG-GFP at postnatal day 21 (n = 5) did not harm the retinal integrity (**G**), while GFP expression was predominantly found along retinal blood vessels (**H**), comparable to AAV2/6-CMV-GFP.

We tested the AAV2/6 serotype on capacity to transduce mouse retinal cells. Wild-type mice (n = 6) received 1 µl of AAV2/6-CMV-GFP (1.5×10^9^ genome copies per injection) at three weeks of age either intravitreally or subretinally. After 21 days, GFP fluorescence was visualized by scanning laser ophthalmoscopy (SLO). Figure 1 shows representative images of the fundus following subretinal or intravitreal injection of the vector (Fig. 1A, C, E, G). The normal retinal architecture was not disturbed by vector administration. Following intravitreal injection in adult mice, GFP expression was detected mainly along the major retinal blood vessels (Fig. 1B). Subretinal injection consistently resulted in transduction of a large but restricted part of the retina (Fig. 1D).

At three months of age, retinas of *Crb1^−/−^* mice show the first signs of degeneration [20], [23], [24]. Structural changes or differences in gene expression patterns might influence the transduction characteristics of AAV2/6-CMV-GFP in the *Crb1^−/−^* retina. However, at three weeks of age, when there is only a very limited amount of retinal degeneration, the results in *Crb1^−/−^* mice (n = 6) were similar to those obtained in wild-type mice (Fig. S1).

To test if injecting at a younger age would increase transduction efficiency, one day old animals (P1) were intravitreally injected with 0.5 µl of AAV2/6-CMV-GFP (0.75×10^9^ genome copies). Transduction in these young animals was found primarily around the optic nerve instead of along major blood vessels (Fig. 1F).

Promoter activity might influence the GFP expression pattern. Hence, we compared the hybrid CMV/β-actin (CAG) promoter [15] with the CMV promoter. The typical transduction pattern along the major blood vessels was also observed following intravitreal injection of AAV2/6-CAG-GFP in three week old mice (1×10^9^ genome copies per injection, Fig. 1H).

### Histological and flow cytometric analysis

**Figure 2:**
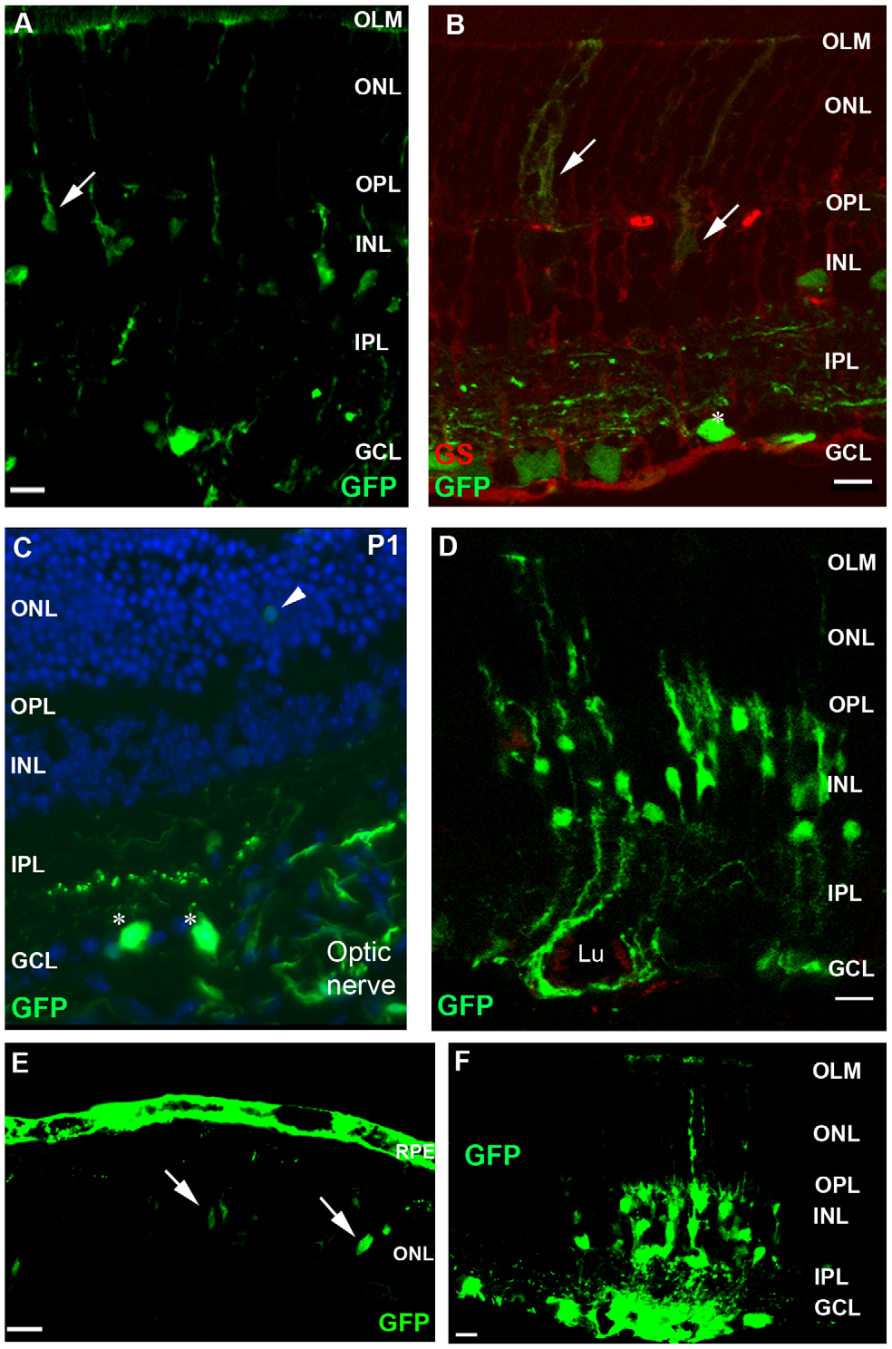
Immunohistochemistry on AAV2/6-CMV-GFP transduced wild-type mouse retinas 3 weeks post injection. Examples of transduced cell types after intravitreal injection of AAV2/6-CMV-GFP at postnatal day 21 (**A**) and confirmed by colocalization of GFP (green) and labeling with the Müller glia cell marker glutamine synthetase (red) (**B**). Transduction of mainly ganglion cells near the optic nerve when AAV2/6-CMV-GFP was injected at postnatal day 1 (**C**). Müller glial cells in the close proximity of a blood vessel (**D**). GFP expression in RPE and photoreceptors cells after injecting AAV2/6-CMV-GFP subretinally (P21, **E**). Transduced cell types found after intravitreal injection of AAV2/6-CAG-GFP at postnatal day 21 (**F**). Symbols/abbreviations used in panels: Arrow; Müller glial cells, Asterisks; ganglion cells, Arrow head; photoreceptor cells, ONL; outer nuclear layer, OPL; outer plexiform layer, INL; inner nuclear layer, IPL; inner plexiform layer, GCL; ganglion cell layer. Lu; vessel lumen. Scale bars represent 10 µm.

**Figure 3:**
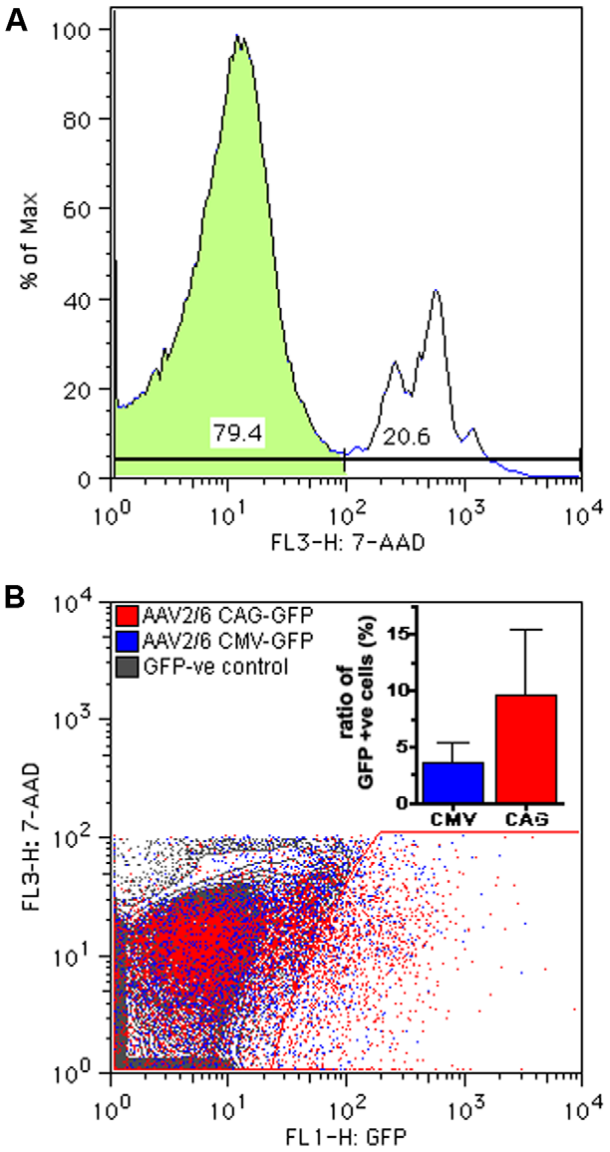
Flow cytometric analysis of AAV2/6-CMV-GFP vs AAV2/6-CAG-GFP transduced retinas. Representative example of 7-AAD staining; the negative (live) population is used for further analysis (**A**). A representative comparison of retinal cells from eyes transduced via intravitreal injection of either AAV2/6-CMV-GFP (blue) or AAV2/6-CAG-GFP (red) resulted in a population of GFP-positive cells. The left edge of the gate for GFP-positive cells was set using non-transduced retinal cells (gray) (**B**). The inset shows the mean (+SEM) of the fraction of the live cells that are GFP-positive in CAG-GFP compared to CMV-GFP eyes (n = 5). These measurements show no statistically significant differences (p>0.6, Mann-Whitney test).

Histological analysis was performed to identify the transduced cell types. Localization of GFP after intravitreal injection of AAV2/6-CMV-GFP at three weeks of age is shown in Figure 2A and revealed the transduction of ganglion cells (asterisk), Müller glial cells (arrow), horizontal, amacrine and bipolar cells (not indicated). Transduced Müller glial cells were easily recognized by morphological characteristics, as these cells extend from the ILM to the OLM of the retina having their cell bodies in the inner nuclear layer (INL). To further corroborate transduction of Müller glial cells (arrow), the sections were stained for the Müller glial cell-specific marker; glutamine synthetase (Fig. 2B). Following intravitreal injection of AAV2/6-CMV-GFP at P1, mainly ganglion cells (asterisk) and sporadically photoreceptor cells (arrow head) close to the optic nerve were found positive for GFP (Fig. 2C). SLO images at three weeks of age showed that transduced cells were mainly present in the proximity of larger blood vessels. The relationship between the blood vessels and transduced cells is depicted in Figure 2D, showing a blood vessel surrounded by transduced Müller glial cells. In contrast, analysis of sections from eyes that had received subretinal injections at three weeks of age revealed that mainly RPE cells were transduced (Fig. 2E). Other transduced cell types, such as photoreceptor cells (arrow heads), could be seen only near the site of injection. Substitution of the CMV for the CAG promoter did not result in an altered transduction pattern (Fig. 2F). Flow cytometric analysis revealed a relatively high variability in the number of transduced cells within the examined retinas, but neither the GFP expression levels nor the number of transduced cells showed statistically significant difference when comparing the CMV promoter with the CAG promoter, using the Mann-Whitney test (Fig. 3A and B).

### Enhancing the transduction efficiency

**Figure 4:**
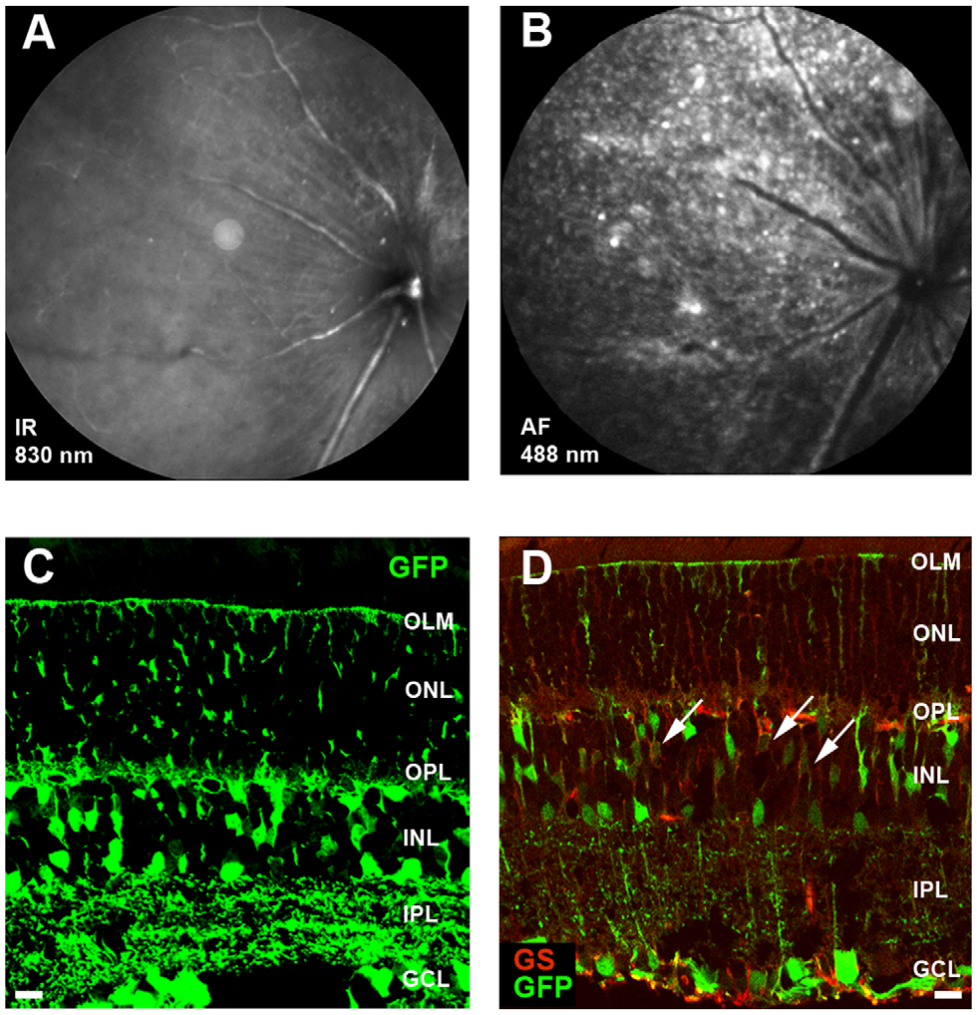
The ILM forms a barrier for efficient Müller glial cell transduction by AAV2/6-CMV-GFP. Representative images of SLO at 830 nm (**A**) and 488 nm (**B**) and of fluorescence microscopy for GFP expression (**C** and **D**) are shown for retinas after intravitreal injection of AA2/6-CMV-GFP in collagenase treated wild-type eyes (n = 4) at three weeks post injection. The integrity of the retinas appeared normal in most cases (**A**). GFP expression was detected throughout the whole retina (**B**). Sections of the retina reveal transduction of Müller glial cells, ganglion, amacrine, horizontal and bipolar cells (**C**). The cell type was confirmed by double labeling with the Müller glial cell marker glutamine synthetase (red) (**D**). Symbols/abbreviations used in panels: Arrow; Müller glial cells, Asterisks; ganglion cells, OLM; outer limiting membrane, ONL; outer nuclear layer, OPL; outer plexiform layer, INL; inner nuclear layer, IPL; inner plexiform layer, GCL; ganglion cell layer. Scale bars represent 10 µm.

**Figure 5:**
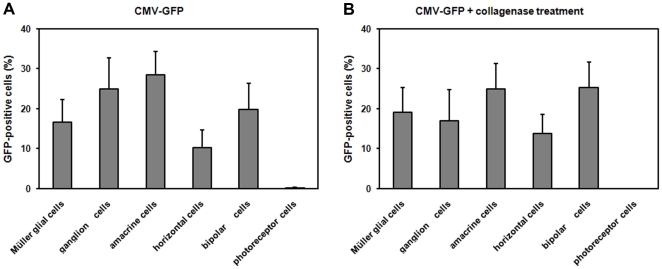
Transduction profiles of AAV2/6-CMV-EGFP retinas with or without collagenase treatement. Representative retinal slices from injected eyes were quantified for the number of each transduced cell type. Specific markers were used to determine the different cell types and to generate histograms comparing tropism profiles with (**B**) and without collagenase treatment (**A**). Transduction efficiencies were calculated based on the ratio of each cell type infected relative to the total number of EGFP-positive cells (n = 9). Error bars represent standard deviation among sample population.

The ILM is thinner at the site of the major retinal blood vessels [25] and is known to be a major obstacle to cell migration after stem cell transplantation from the vitreal side of the retina [26]. This phenomenon might explain the typical transduction pattern along the major blood vessels observed following intravitreal administration of AAV2/6 viral particles. To overcome this barrier, AAV2/6-CMV-GFP was intravitreally administrated after disruption of the ILM by collagenase VII treatment [27]. The integrity of the retinas was studied by SLO (Fig. 4A), which revealed acute retinal hemorrhage in 2 out of 4 wild-type and all *Crb1^−/−^* retinas (data not shown). While the left eye of both wild-type and *Crb1^−/−^* mice was left untreated and showed the same transduction pattern as depicted in Figure 1B, the collagenase-treated right eye was much more efficiently transduced (Fig. 4B). Increased transduction was observed in all wild-type and *Crb1^−/−^* retinas tested (n = 4 and n = 5, respectively). Immunohistochemical analysis showed GFP expression throughout the retina, with expression in many retinal cell types of the inner retina but not in photoreceptor cells (Fig. 4C). We confirmed the transduction of Müller glial cells by co-staining with antibodies against glutamine synthetase (Fig. 4D). Transduction profiles were established using cell type specific markers (for horizontal cells we used Prox1, and for ganglion and amacrine cells calretinin) comparing transduced retinas with and without collagenase treatment (Fig. 5A and B). No significant changes in the transduction profile were found after collagenase treatment.

### Transduction of the human retina

**Figure 6:**
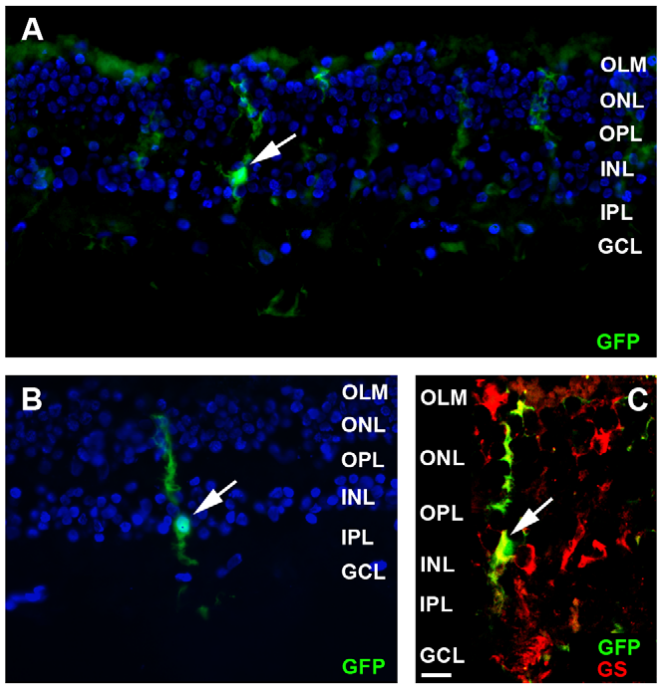
Transduction of human cultured retinas with AAV2/6-CMV-GFP. Retinas were prepared from donor eyes and transduced with AA2/6-CMV-GFP, by injection of 1 µl viral particle suspension under the ILM. Retinas were cultured for 7 days (n = 2), followed by fluorescence microscopic analysis of cryostat sections (10 µm). GFP-positive Müller glial cells (green signals) were found near the injection site (**A**) as well as further away (**B**). The cell type was confirmed by double labeling with the Müller glial cell marker glutamine synthetase (red) (**C**). Symbols/abbreviations used in panels: Arrow; Müller glial cells, OLM; outer limiting membrane, ONL; outer nuclear layer, OPL; outer plexiform layer, INL; inner nuclear layer, IPL; inner plexiform layer, GCL; ganglion cell layer.

Our ultimate goal is to develop gene therapy to treat human eye diseases. In order to test whether the AAV2/6 vectors are able to transduce human retinal cells, we obtained donor eyes from the Cornea Bank Amsterdam within 48 hours post mortem and prepared the human retina for culture. The ILM was lifted and AAV2/6-CMV-GFP particles (1×10^9^ genome copies) were injected between the ILM and the neural retina. After 7 days of culture, GFP expression could be detected as a halo around the injection site. When the retinas were sectioned, GFP expression was observed in Müller glial cells near the injection site (Fig. 6A) as well as further away (Fig. 6B). We confirmed the cell type by co-staining with antibodies against glutamine synthetase (Fig. 6C). These results show for the first time that not only mouse but also human Müller glial cells can be transduced by AAV2/6.

### Restricted transgene expression in activated Müller glial cells

CRB1 is a protein expressed in Müller glial cells [17] and mutations in the *CRB1* gene cause LCA. Previously, we evaluated the *Crb1^−/−^* mouse as model for CRB1 related eye diseases [20]. Retinal degeneration in *Crb1^−/−^* mice was observed in only one quadrant of the retina and occurred in foci. Müller glial cells aligning these foci were activated and expressed GFAP.

The AAV2/6 vectors tested so far in this study do not exclusively target Müller glial cells, but display tropism for other neural components of the inner retina (bipolar, amacrine, horizontal and ganglion cells). Hence, to obtain restricted Müller glia cell transgene expression, we tested the mouse GFAP promoter [16] in our mouse model for progressive Müller cell gliosis, using AAV2/6-GFAP-GFP viral particles. Intravitreal injection of these viral particles (8×10^9^ genome copies) in *Crb1^−/−^* mice was performed at three weeks of age, when signs of retinal degeneration were still very limited [20]. Importantly, in an unaffected wild type or *Crb1^−/−^* retina, Müller glial cells are not activated and will not express GFAP.

**Figure 7:**
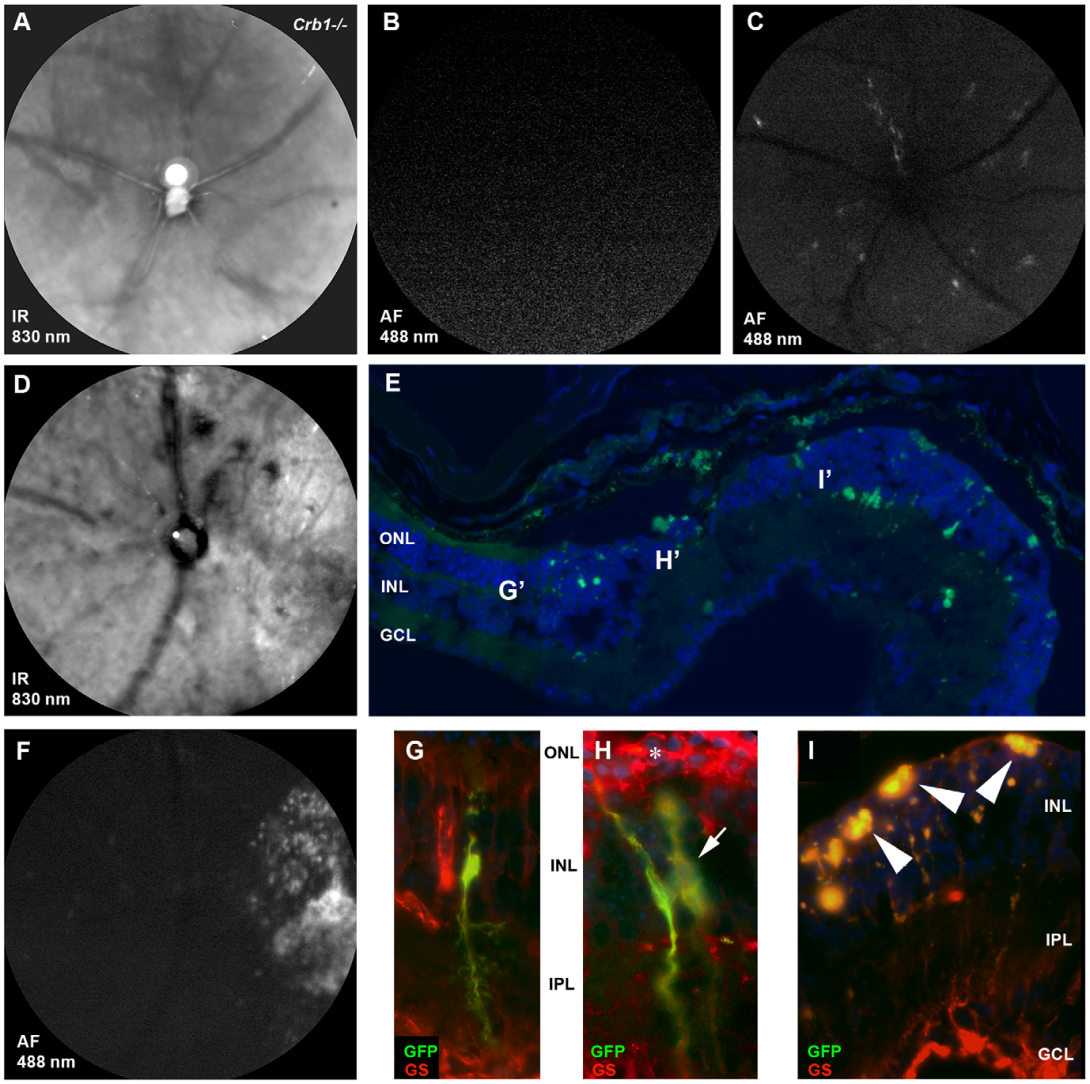
Light-induced GFP expression in AAV2/6-GFAP-GFP transduced *Crb1^−/−^* Müller glial cells. *Crb1^−/−^* mice, three weeks of age, were intravitreally injected with AAV2/6-GFAP-GFP (8×10^9^ genome copies; n = 3). At 21 days after injection, before light exposure, retinal degeneration and fluorescence were analyzed by SLO at 830 nm (**A**) and 488 nm (**B**), respectively. 10 weeks after transduction the mice were exposed to white light (3000 lux) for 72 hours. GFAP-driven GFP expression was detected by SLO in *Crb1^−/−^* mice (n = 7) (**C**). One out of seven *Crb1^−/−^* animals showed severe retinal degeneration on SLO analysis as reported before [20], [24] (**D**). The retinas showed extensive fluorescence in one quadrant of the retina (**F**). In the severely affected retina (**E**), most GFP and glutamine synthetase positive Müller glial cells were found at the border of the area of ONL degeneration (**G** and **H**). Slices adjacent to the one showing the overview in E were subjected to immunohistochemistry with antibodies against glutamine synthetase. G', H' and I' are the positions of the figures G, H and I taken from adjacent slices to the overview shown in E. The asterisk in (**H**) points at the abnormal structure of the activated Müller glial cells and glial scar formation. In the middle of the degenerative area, the ONL is completely lost (**E**). In this area, highly auto-fluorescent patches of cell debris, indicated by arrowheads (**I**), accounted for most of the fluorescent signal found on SLO. Symbols/abbreviations used in panels: Arrow; Müller glial cells, ONL; outer nuclear layer, INL; inner nuclear layer, IPL; inner plexiform layer, GCL; ganglion cell layer.

Previously, we described that the *Crb1^−/−^* phenotype is enhanced when the retina is exposed for 72 hours to white light (3000 lux) [20]. The induction of the *Crb1^−/−^* phenotype was accompanied by activated Müller glial cells that were positive for GFAP, a phenomenon under the same conditions not observed for wild type Müller glial cells. The same strategy of light exposure was used on *Crb1^−/−^* mice following intravitreal injection of AAV2/6-GFAP-GFP (8×10^9^ genome copies; n = 7). At 10 weeks after injection, the animals were exposed to white light for 72 hours. No abnormalities in the retina were observed before light exposure (Fig. 7A) as reflected by the absence of GFAP-driven GFP expression at 21 days after injection (Fig. 7B). After light exposure, disturbances occurred in the *Crb1^−/−^* retinas (n = 7) but not in wild type controls (n = 5). When these *Crb1^−/−^* animals were examined by SLO, four out of seven animals exhibited GFP-positive spots aligning the major retinal blood vessels or the injection site (Fig. 7C). A severe form of retinal degeneration resulting in the loss of retinal integrity detectable by SLO was observed in one of seven *Crb1^−/−^* animals analyzed (Fig. 7D). Histological analysis confirmed a completely disturbed and degenerated outer nuclear layer (ONL; Fig. 7E). Characteristic for the *Crb1^−/−^* phenotype in mice, only one quadrant of the retina was affected (Fig. 7F) [20]. GFP-positive Müller glial cells could be detected, especially at the border of the degenerated area (Fig. 7G and H). Müller glial cells in the affected area were not only activated but also disorganized (Fig. 7H, asterisk). It should be noted though that the observed fluorescence in the SLO originates not only from the activated Müller glial cells, but mostly from auto-fluorescent signals from deposited cell debris in the disturbed areas of the ONL (Fig. 7I, arrowheads). This auto-fluorescence could be distinguished from GFP expression by fluorescence microscopy of cryostat sections.

**Figure 8:**
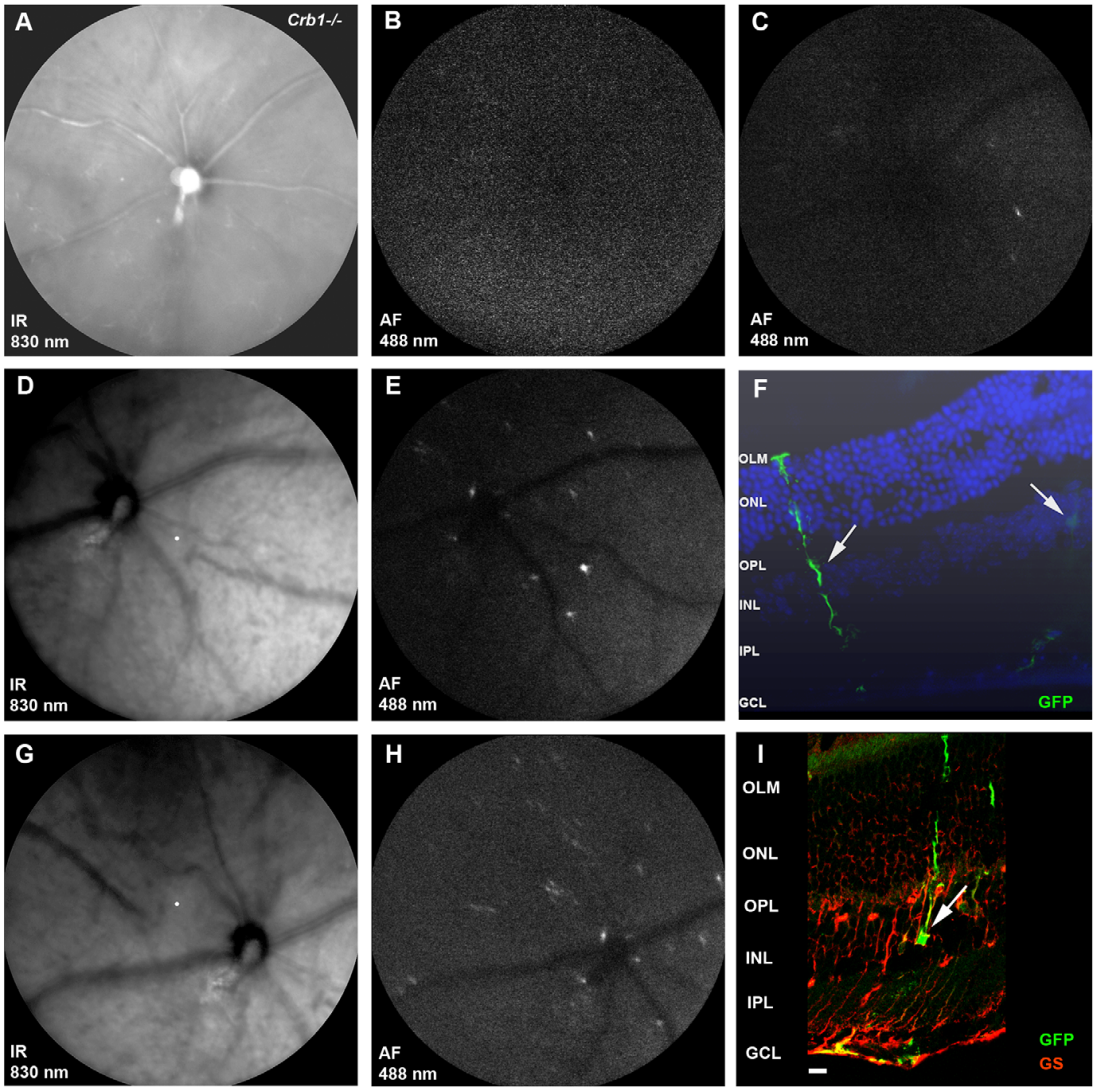
CNTF-induced GFP expression in AAV2/6-GFAP-GFP transduced Müller glial cells. 3-week-old *Crb1^−/−^* mice were intravitreally injected with AAV2/6-GFAP-GFP (8×10^9^ genome copies; n = 3). Three weeks after injection, the integrity of the retina appeared normal on SLO (**A**). No GFP signal was observed (**B**). At 28 days after transduction, eyes were re-injected intravitreally with CNTF. GFAP-driven GFP expression was detected by SLO at one day (**C**) and four days (**E** and **H**) after administration of CNTF. Secondary injections did not affect the retinal integrity (**D** and **G**). Activated Müller glial cells expressing GFP were found on sections (10 µm) of the CNTF challenged eyes (**F**) and immuno-histochemistry confirmed the cell type as colocalization with glutamine synthetase was demonstrated (**I**). Symbols/abbreviations used in panels: Arrow; Müller glial cells OLM; outer limiting membrane, ONL; outer nuclear layer, OPL; outer plexiform layer, INL; inner nuclear layer, IPL; inner plexiform layer, GCL; ganglion cell layer.

Due to the strong variability in the *Crb1^−/−^* phenotype we decided to test the GFAP promoter in a different way. At four weeks after the intravitreal injection with AAV2/6-GFAP-GFP the integrity of the retina was still intact (Fig. 8A) and no fluorescent signal could be detected using SLO (Fig. 8B). GFAP expression in Müller glial cells was induced by an intravitreal injection of CNTF at 28 days after transduction [16], [28]. At one day after CNTF injection a few spots of fluorescence were observed with SLO (Fig. 8C). While the integrity of the retina was still not compromised (Fig. 8D and G), more signal was seen at four days after injection (Fig. 8E and H), In sections, GFP-positive Müller glial cells were detected aligning the major retinal blood vessels (Fig. 8F). Importantly, no GFP-positive ganglion, amacrine, bipolar, horizontal, or photoreceptor cells were detected. Thus, in contrast to GFP expression found in multiple retinal cell types following transduction with an AAV2/6 vector that contains a CMV promoter (Fig. 2A), AAV2/6-GFAP-GFP targets transgene expression specifically to activated Müller cells. The cells expressing GFP were indeed Müller glial cells since they were positive for glutamine synthetase (Fig. 8I). The number of GFP expressing cells is quite limited due to the fact that only Müller glial cells in the proximity of major retinal blood vessels were transduced with AAV2/6-GFAP-GFP of which only some became activated by CNTF. These results suggest that AAV2/6 vectors carrying the GFAP promoter are suitable for expressing genes of interest specifically in Müller glial cells close to retinal blood vessels at the site of retinal degeneration. Furthermore, the transgene will only be expressed in Müller glial cells when they become activated.

## Discussion

Here we showed that, retinal ganglion, amacrine, bipolar and Müller glial cells in the proximity of major retinal blood vessels were transduced by AAV2/6-CMV-GFP and showed reporter gene expression. Additionally, using AAV2/6-GFAP-GFP, we showed stress- or injury-dependent GFAP-driven gene expression limited to activated Müller glia cells aligning the major retinal blood vessels.

In most, if not all, retinopathies, independent of the underlying cellular defect Müller glial cells become activated [1]. Since they span the entire retina and align the sites of injury, activated Müller glia cells might be the optimal “factory” for secreted proteins that protect the surrounding neurons [29]–[31]. In this study, we aimed to generate an AAV-based vector system to specifically express transgenes in activated Müller glial cells. By using AAV2/6 containing the Müller glial cell-specific GFAP promoter, we achieved transgene expression that is selectively targeted to activated Müller glial cells aligning the major retinal blood vessels. We thereby aligned transgene expression with affected areas of the retina, in a mouse model of retinal degeneration with extensive Müller glial cell activation.

AAV2/6-mediated transduction of rat Müller glial, ganglion, bipolar and amacrine cells has previously been demonstrated [12], but the expression was not reported to be limited to Müller cells aligning the major blood vessels. Here, we set out to optimize AAV2/6-mediated transduction of mouse Müller glial cells. Following intravitreal administration in the adult mouse, AAV2/6-CMV-GFP was able to transduce Müller glial, ganglion, bipolar, horizontal and amacrine cells aligning the blood vessels. In a clinical setting, intravitreal injections would be the preferred route of administration for several reasons. Firstly, intravitreal administration would be potentially less deleterious to an already weakened and diseased retina [32], [33]. Secondly, viral particles can spread more easily when delivered via the intravitreal route allowing a wider area of retinal transduction. Surprisingly, transduction by AAV2/6 was mainly seen along the major retinal blood vessels. As for other AAV serotypes [13] and stem cells [26], the ILM proved to be a barrier for AAV2/6 penetration into the retina. Since the human ILM is much thicker we decided to lift the ILM of human retina explants and apply the viral vectors under the ILM. Following this procedure we were able to transduce human Müller glial cells with AAV2/6. While collagenase treatment is not applicable to patients, ILM peeling or lifting is [34] and might be used as a way to administer AAV2/6 to the human retina in order to transduce human Müller glial cells. Hata et al [35] demonstrated that the intravitreal route and the subretinal route are not harmful to the function and appearance of the retina. Moreover, they showed that ILM peeling in the monkey eye is a way to administer drug without deleterious effects on the appearance of the retina. In this study, we did not assess whether *in vivo* AAV2/6 transduces human Müller glia cells aligning the major blood vessels, as is the case in the mouse retina. Expression limited to Müller glia cells aligning the major blood vessels could however be of potential advantage for expressing drugs locally that currently are systemically administered. E.g. Müller glia cells could be used to express and secrete VEGF inhibitors in the retina of patients with age related macula degeneration, or other drugs that prevent neovascularization in the retina of patients with retinal dystrophy. Whilst we used AAV2/6 to transduce Müller glial cells aligning the major blood vessels, recently other AAV capsids generated via directed evolution have been shown to be more potent transducers of rat astrocytes and retinals cells [36]. One AAV6 variant (ShH10) showed efficient and selective transduction of rat Müller cells throughout the retina, not limited to Müller cells aligning the major blood vessels [37]. Future experiments might demonstrate their potential to transduce human Müller glial cells.

GFAP is a intermediate filament protein present in Müller glial cells, astrocytes, microglia, and Schwann cells that is upregulated in activated cells in reaction to injury [1]. GFAP is not only upregulated, but also actively involved in the morphological changes of Müller glial cells that follow experimental retinal detachment [33] and age-related macular degeneration (AMD) [38]. Even retinal detachment caused by a subretinal injection gave rise to activation of Müller glial cells as reflected by detectable GFAP expression levels at 28 days after injecting a balanced salt solution [39]. The GFAP promoter appeared effective in expressing GFP as transgene in activated Müller glial cells of the degenerating rat retina, when delivered subretinally by lenti-viral vectors [40]. Here, we delivered the GFAP promoter and GFP transgene to mouse Müller glial cells aligning the major retinal blood vessels via intravitreal injection of AAV2/6-GFAP-GFP. The advantage of the intravitreal administration route is the absence of Müller glial cell activation [28] as was confirmed by the absence of fluorescent signal at 21 days after injection of AAV2/6-GFAP-GFP.

We demonstrated for the first time that AAV transgene expression via the stress-inducible GFAP-promoter allows endogenous modulation. Müller glial cells were activated using two different approaches: intravitreal administration of ciliary neurotrophic factor (CNTF) and light exposure to CRB1 deficient mice. The yield of the activated Müller glial cells expressing the transgene was low due to the fact that only 3% of the total retinal cells were transduced by intravitreal injection of AAV2/6 without removing the ILM (Fig. 3). Müller glial cells make up only 15% of the transduced cells (Fig. 5) and with 3 days of light exposure or 4 days after CNTF administration not all transduced Müller glial cells became activated [20], [28]. Different approaches as ILM peeling or lifting in a human setting or using the AAV6 variant (ShH10) [37] to deliver the GFAP driven transgene might increase the yield of activated Müller glial cells expressing the transgene.

Mice lacking CRB1 showed upregulation of GFAP before onset of retinal disorganization and degeneration [20]. For gene therapy using trophic factors (e.g. GDNF), it might be very important that the therapeutic transgene is not continuously expressed but modulated along with severity of the pathology. This requirement might be fulfilled using the GFAP promoter in the AAV vector to drive the transgene expression in activated Müller glial cells. The AAV2-GFAP vector system might now be used to deliver various molecules to retinas with different types of disease or injury to test their therapeutic potency.

## Materials and Methods

### Animals

All procedures concerning animals adhered to the ARVO statement for the use of animals in ophthalmic and vision research, and were performed with permission of the animal experimentation committee (DEC) of the Royal Netherlands Academy of Arts and Sciences (KNAW), permit number NIN08-17. The generation of the *Crb1^−/−^* mice was described previously [20]. All mice used were maintained on a 50% C57BL/6 and 50% 129/Ola genetic background. Except for the light-exposed mice, animals were maintained on a 12 h day/night cycle and supplied with food and water *ad libitum.*

### Generation and purification of the viral vectors

The plasmids used to generate the AAV vectors were derived from plasmid pTRCGW and consist of the inverted terminal repeats (ITRs) of AAV2, the CMV, CAG or GFAP promoter, the cDNA encoding GFP, the simian virus 40 (SV40) polyadenylation signal and the woodchuck post-transcriptional regulatory element. The CMV promoter in the original plasmid was replaced by a 2.6 kb mouse GFAP promoter fragment [16]. AAV stocks were generated and purified as described previously [41]. Briefly, plasmids containing the transgene flanked by the ITRs were co-transfected with the AAV2/6 helper plasmid pDP6 (Plasmid Factory, Bielefeld, Germany) into HEK293T cells to generate cross-packaged AAV2/6 viral vectors [12], [36]. At the third day after transfection, the medium was changed for lysis buffer (50 mM Tris, 2 mM MgCl_2_, 150 mM NaCl and 0.1% Triton X-100). After DNAse treatment, the crude lysate was loaded onto an iodixanol density gradient (Sigma, St Louis, Mo, USA) and centrifuged for 70 min in a Beckman XL-100K ultracentrifuge at 69000 rpm at 16°C [42]. Fractions containing the viral vectors were collected and concentrated using Amicon Ultra-15 concentrators. All viral titers were determined by q-PCR and all viral stocks with titers above 1×10^12^ genome copies/ml were stored at −80°C until use.

### Intravitreal and subretinal injections

Three-week-old mice were anesthetized with 100 mg/kg ketamine and 5 mg/kg xylazine i.p. Their pupils were dilated with eye drops that contain 5 mg/ml tropicamide (Chauvin Benelux, Brussels, Belgium). To prevent dehydration of the cornea, the eyes were treated with viscotears (Novartis Pharma, Arnhem, The Netherlands). The mice were placed on a heating pad to maintain their body temperature at 37°C during the whole procedure. Subsequently, 1.5×10^9^ genome copies of vector in 1 µl were either subretinally or intravitreally injected using a 33 gauge needle (Hamilton, Bonaduz, Switzerland). The mice were allowed to recover and their eyes were treated with 10 mg/g chloramphenicol (Ratiopharm, Zaandam, The Netherlands) to prevent infections. Intravitreal injections were done with and without disruption of the ILM by collagenase treatment [27]. For collagenase treatment, mice were anesthetized by inhalation of isoflurane (5%). Following dilation of their pupils, 1 µl of collagenase type VII (50 units/ml; Sigma-Aldrich, Zwijndrecht, The Netherlands) was intravitreally injected into the right eye. The left eye was not injected. Seven hours after the collagenase injection, the mice were anesthetized again (ketamine and xylazine). Their pupils were dilated and 0.5×10^9^ genome copies of vector in 1 µl containing laminin and α2-macroglobulin (1 mg/ml each; both Sigma-Aldrich) were intravitreally injected into both eyes.

Mice intravitreally injected with viral vectors carrying the GFAP promoter were either additionally intravitreally injected with 0.5 µl of 1 mg/ml CNTF at 28 days after viral transduction or exposed to white light at 10 weeks after viral transduction.

### Light exposure

The animals were placed in a white box and continuously exposed to diffuse white light of 3000 lux (TLD-18W/33tubes, Philips; 350-700 nm) for 72 h without pupillary dilation. Immediately after these 72 h of light exposure the animals were anesthetized and subjected to scanning-laser ophthalmoscopy (SLO).

### Scanning-laser ophthalmoscopy

Three weeks after injection of the viral vector, the mice were anesthetized by ketamine and xylazine i.p. Dilation of their pupils was done with eye drops containing 50 mg/ml phenylephrine HCl (Théa Pharma, Zoetermeer, The Netherlands). To prevent dehydration of the cornea, the eyes were treated with Methocel (CIBA Vision, Breda, The Netherlands). SLO was performed with the Heidelberg Retina Angiograph 2 (HRA 2; Heidelberg Engineering, Heidelberg, Germany) as described previously [43]. A solid state laser at 488 nm was used to excite GFP. Separation of excitation and fluorescent light was done with a barrier filter at 500 nm. Infrared reflectance images were generated using a diode laser at 830 nm.

### Immunohistochemical analysis, fluorescence microscopy and cell counting

The anaesthetized mice were sacrificed by cervical dislocation immediately after SLO imaging. Their eyes were collected, fixed for 30 min in 4% paraformaldehyde in PBS (pH 7.4) and cryoprotected by subsequent incubations of 30 min in 5 and 30% sucrose. After cryoprotection, the eyes were embedded in Tissue-Tek (Sakura, Zoeterwoude, The Netherlands), frozen and stored at −80°C. Sections of 7 µm were generated with a Leica CM3050 cryostat (Leica Microsystems, Rijswijk, The Netherlands), mounted onto SuperFrost Plus glass slides (Menzel-Gläser, Braunschweig, Germany) and air-dried. The sections were either enclosed immediately in Mowiol 4–88 (Sigma-Aldrich) supplemented with Hoechst 33258 (Invitrogen, Breda, The Netherlands) to counter stain the cell nuclei or used for immunohistochemical analysis. Sections used for immunohistochemical analysis [44] were rehydrated in PBS and blocked for 1 h in 10% normal goat serum (Jackson ImmunoResearch, Suffolk, UK), 0.4% Triton X-100 and 1% bovine serum albumin (BSA) in PBS. The sections were then incubated overnight (4°C) with mouse anti-glutamine synthetase IgGs (1∶300; BD Biosciences, Breda, The Netherlands) or anti-GFP IgGs (1∶300; Chemicon, Hampshire, UK) or rabbit anti-Prox1 (1∶500; ABCam Ltd, Cambridge, U.K.), or rabbit anti-calretinin (1∶500; ABCam Ltd, Cambridge, U.K.) diluted in 0.3% normal goat serum, 0.4% Triton X-100 and 1% BSA in PBS. After washing the sections three times with PBS, they were incubated for 1 h with Cy3-conjugated goat anti-mouse IgGs (Jackson ImmunoResearch) diluted in 1% BSA in PBS. The sections were washed three times with PBS and enclosed in Mowiol 4–88 with Hoechst 33258. The GFP as well as the Cy3 signal was visualized with a Leica DMRE fluorescence microscope (Leica Microsystems).

Transduction profiles were analyzed by counting individual GFP-positive cells, defined by their typical morphology and by co-labeling with Hoechst 33258 (Invitrogen) and anti-glutamine synthetase or anti-Prox1 or anti-calretinin. At least three different sections from each eye within three independent experiments (n = 9/group) were counted at ×40 magnification. The number of GFP-positive cells was divided by the total number of cells present in the same ×40 magnified area to obtain the percentage of transduced cells. The percentage of transduced cells from each group was then averaged and standard errors were calculated.

### Flow cytometric analysis

Neural retina was isolated from enucleated eyes by dissection of the anterior part of the eye and attached vitreous from the eye cup, following a transscleral incision. Individual retinae were incubated in 1.2 ml of enzyme incubation buffer (4 g/l MgCl_2_, 2.55 g/l CaCl_2_, 3.73 g/l KCl, 8.95 g/l NaCl, pH 6–7) containing 92.5 U Collagenase I (Worthington, Lakewood NJ, USA) and 0.25 mg DNase I (Roche, Mannheim, Germany) for 1 hour at 37°C. The tissue was triturated every 10–12 minutes using a Gilson p1000 pipet and tips with decreasing bore size. Cell suspensions were washed in 20 ml cell suspension buffer (8 g/l NaCl, 0.4 g/l KCl, 1.41 g/l NaHPO_4_ anhydrous, 0.69 g/l NaH_2_PO_4_.H_2_O, 2 g/l D-glucose, pH 7.4, containing 0.2% BSA) and cells recovered in a pellet following centrifugation at 1,700×g for 10 minutes. For flow cytometric analysis an equivalent of a tenth of one retina was used per staining procedure. Cell suspensions were stained by adding 2.5 µl 7-AAD solution (BD Biosciences, San Jose CA, USA) to 100 µl cell suspension 10 minutes before analysis. Flow cytometric analysis was performed using a FACSCalibur (BD Biosciences), and data was analyzed using FlowJo (Mac V8.8.6; Tree Star, Ashland OR, USA). Statistical analysis of the data was performed with Prism 4.0 for Macintosh, using the Mann-Whitney test for the analysis of the percentages of GFP positive cells of the 7-AAD negative population, and the mean fluorescence intensity.

### *In vitro* transduction of the human retina

Human donor eyes were acquired from the Dutch Cornea Bank Amsterdam and were processed within 48 h after death. Retinas were dissected free from the posterior eyecup and placed in a sterile dish. The vitreus was removed as much as possible using scissors. The ILM was then lifted with a 20ga V-Lance® hook (Alcon laboratories, Forth Worth, TX, USA) and 1 µl containing 1×10^9^ genome copies of AAV2/6-CMV-GFP was injected under the ILM. The retina was cultured for 7 days according to the previously described protocol for culturing mouse retina explants [17]. After 7 days, the retinas were fixed for 30 min in 4% paraformaldehyde in PBS (pH 7.4) and cryoprotected by subsequent incubations of 30 min in 5 and 30% sucrose. They were stored at −80°C until further use.

## Supporting Information

Figure S1 SLO of AAV2/6-CMV-GFP transduced *Crb1^−/−^* mouse retina at 3 weeks post injection. After intravitreal injection of AAV2/6-CMV-GFP at postnatal day 21 (n = 6), the integrity of the retina appeared normal (A). The fluorescent signal for GFP was detected along the major retinal blood vessels (B). After subretinal injection of AAV2/6-CMV-GFP at postnatal day 21 (n = 6), the integrity appeared normal (C). GFP fluorescence was detected along the major retinal blood vessels (D).
(2.32 MB TIF)
