# Supplementary figures and images for: GFAP-Driven GFP Expression in Activated Mouse Müller Glial Cells Aligning Retinal Blood Vessels Following Intravitreal Injection of AAV2/6 Vectors
Source: PLoS One. 2010 Aug 24;5(8):e12387. doi: 10.1371/journal.pone.0012387 (PMC2927518; doi:10.1371/journal.pone.0012387)

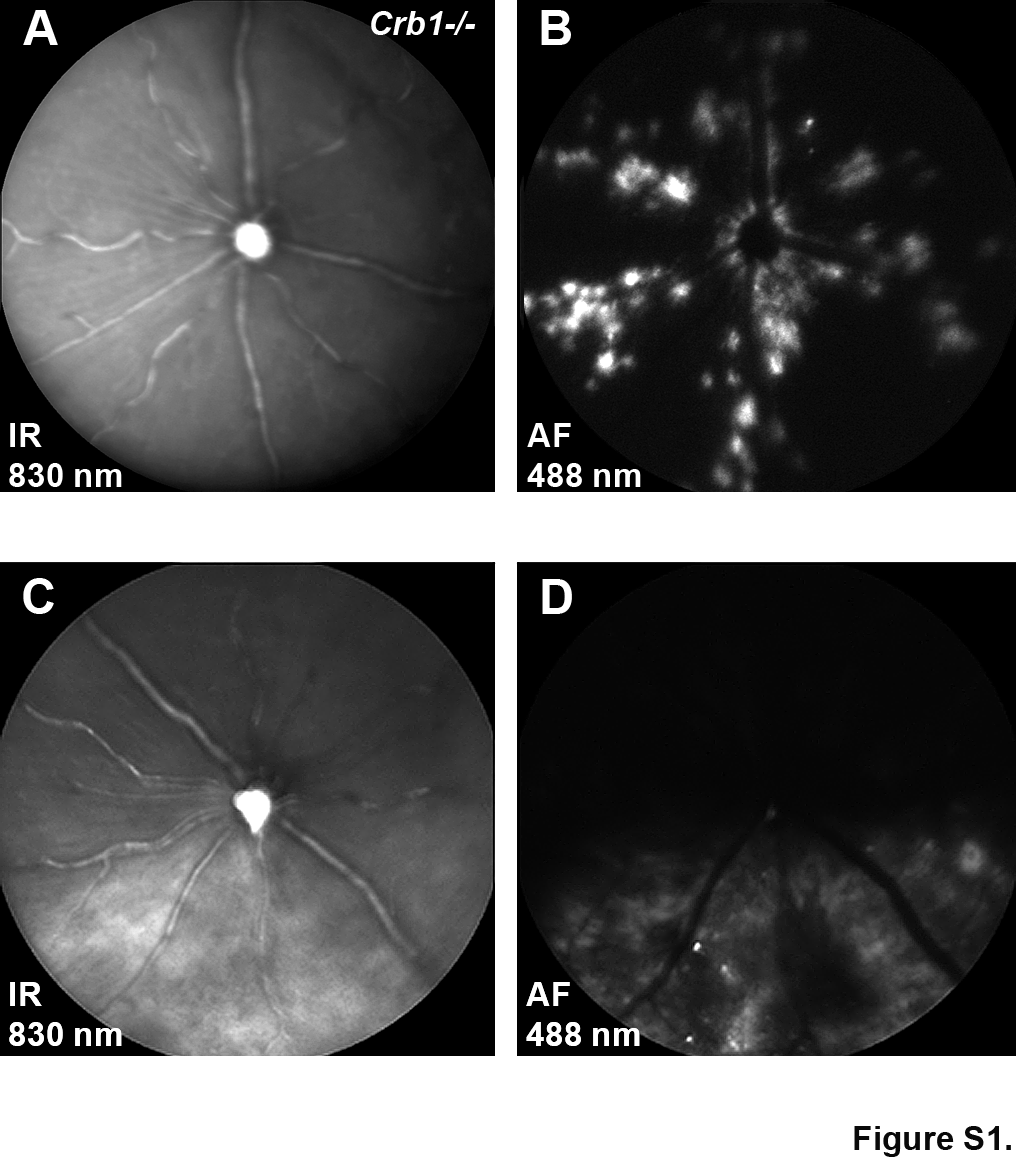

Supplement: Figure S1 — SLO of AAV2/6-CMV-GFP transduced Crb1−/− mouse retina at 3 weeks post injection. After intravitreal injection of AAV2/6-CMV-GFP at postnatal day 21 (n = 6), the integrity of the retina appeared normal (A). The fluorescent signal for GFP was detected along the major retinal blood vessels (B). After subretinal injection of AAV2/6-CMV-GFP at postnatal day 21 (n = 6), the integrity appeared normal (C). GFP fluorescence was detected along the major retinal blood vessels (D). (2.32 MB TIF) [file pone.0012387.s001.tif]
